# The feasibility of extracorporeal cardiopulmonary resuscitation for patients with active cancer who undergo in-hospital cardiac arrest

**DOI:** 10.1038/s41598-022-05786-8

**Published:** 2022-01-31

**Authors:** Yo Sep Shin, Pil-Je Kang, Youn-Jung Kim, Seung Mok Ryoo, Sung-Ho Jung, Sang-Bum Hong, Won Young Kim

**Affiliations:** 1 Department of Emergency Medicine, Asan Medical Center, University of Ulsan College of Medicine, 88 Olympic-ro 43-gil, Songpa-gu, Seoul, 05505 Korea; 2 Department of Thoracic and Cardiovascular Surgery, Asan Medical Center, University of Ulsan College of Medicine, Seoul, Korea; 3 Department of Pulmonary and Critical Care Medicine, Asan Medical Center, University of Ulsan College of Medicine, Seoul, Korea

**Keywords:** Cancer, Cardiology, Oncology

## Abstract

Indications of extracorporeal cardiopulmonary resuscitation (ECPR) are still debatable, particularly in patients with cancer. Prediction of the prognosis of in-hospital cardiac arrest (IHCA) in patients with cancer receiving ECPR is important given the increasing prevalence and survival rate of cancer. We compared the neurologic outcomes and survival rates of IHCA patients with and without cancer receiving ECPR. Data from the extracorporeal membrane oxygenation registry between 2015 and 2019 were used in a retrospective manner. The primary outcome was 6-month good neurologic outcome, defined as a Cerebral performance category score of 1 or 2. The secondary outcomes were 1- and 3-month good neurologic outcome, and 6-month survival. Among 247 IHCA patients with ECPR, 43 had active cancer. The 6-month good neurologic outcome rate was 27.9% and 32.4% in patients with and without active cancer, respectively (*P* > 0.05). Good neurologic outcomes at 1-month (30.2% vs. 20.6%) and 3-month (30.2% vs. 28.4%), and the survival rate at 6-month (39.5% vs. 36.5%) were not significantly different (all *P* > 0.05) Active cancer was not associated with 6-month good neurologic outcome by logistic regression analyses. Therefore, patients with IHCA should not be excluded from ECPR solely for the presence of cancer itself.

## Introduction

With the implementation of extracorporeal cardiopulmonary resuscitation (ECPR) as a rescue therapy for patients with refractory cardiac arrest becoming widespread, both the survival rate and neurologic outcome have steadily increased over the last decades^1–4^. In patients who undergo refractory cardiac arrest, ECPR assists with rapid restoration of perfusion, leading to improved survival^5,6^. However, ECPR has only been adopted for selected patients as recommended by the American Heart Association guidelines due to its high cost and need for many trained personnel^7,8^.

Meanwhile, as the survival rate of patients with cancer has increased, so has the rate of admission due to the intensive and continued care of patients, and some eventually suffer from in-hospital cardiac arrest (IHCA)^9–11^. Despite the fact that patients with cancer account for 14% of patients with IHCA, according to the study^12^, they receive less intensive treatment because of the risk of acquired coagulopathy and the uncertainty of the long-term outcomes; furthermore, their prognosis is poor compared to those without cancer^13–15^.

Although cancer is traditionally considered as one of the contraindications for extracorporeal membrane oxygenation (ECMO)^16^, considering the increasing survival rate and the fact that patients with cancer represent a considerable portion of IHCA cases, it is reasonable to consider implementing ECPR in these cases, particularly based on advance directives and anticipated life expectancy. While some studies have shown increased survival of patients with cancer using ECMO or extracorporeal life support (ECLS), the majority are targeted to paediatric patients, hematologic malignancies, or acute respiratory failure without cardiac arrest^17–21^; therefore, it remains unclear whether ECPR could benefit patients with cancer who suffer IHCA.

In the current study, we investigated the neurologic outcomes and survival rates of adult patients with cancer who underwent IHCA and who received ECPR; the outcomes of these patients were compared with those of adults without cancer who suffered IHCA and received ECPR.

## Results

### Baseline characteristics

Using data from the ECMO registry, 977 patients from January 2015 to December 2019 were reviewed. Among these patients, we excluded 462 who only received ECMO and not ECPR, 229 patients who received veno-veno ECMO, and 39 patients who received ECPR in an OHCA setting. Finally, 247 patients were included for final analysis; among them, 43 patients had active cancer at the time of arrest, and the remaining 204 patients had no cancer. And the details in patients with active cancer are demonstrated in supplementary Fig. 1.

The characteristics of the subjects, including age, sex, obesity, comorbidities, and laboratory data on the day of arrest, are listed by group (active cancer and cancer-free) in Table 1. The prearrest CPC score, proportion of male sex, obesity, laboratory findings on the day of arrest (except for creatinine), and medical history, including cardiac arrest, acute myocardial infarction, hypertension, diabetes, cerebrovascular accident, liver cirrhosis, and transplantation, were not significantly different between the two groups. The patients with active cancer were significantly older, and the proportion of angina, arrhythmia, heart failure, percutaneous coronary intervention (PCI), coronary artery bypass graft (CABG), and chronic kidney disease (CKD) was higher in cancer-free patients (*P* < 0.05).

**Table 1:** Baseline characteristics.

Variables	Total (n = 247)	Patients with active cancer (n = 43)	Patients without active cancer (n = 204)	*P*–value
Age, years*	61 (51–72)	65 (57–73)	60 (50–69)	0.023
Sex, male (%)	179 (72.5)	34 (79.1)	145 (71.1)	0.286
Obesity (BMI > 25 kg/m^2^) (%)	86 (34.8)	16 (37.2)	70 (34.3)	0.717
Prearrest CPC score	1.60 (0.64)	1.65 (0.61)	1.59 (0.64)	0.556
**Medical history (%)**				
Cardiac arrest	16 (6.5)	1 (2.3)	15 (7.4)	0.319
AMI	29 (11.7)	2 (4.7)	27 (13.2)	0.112
Angina	54 (21.9)	3 (7.0)	51 (25.0)	0.009
Arrhythmia	36 (14.6)	2 (4.7)	34 (16.7)	0.042
HF	50 (20.2)	1 (2.3)	49 (24.0)	0.001
PCI	43 (17.4)	3 (7.0)	40 (19.6)	0.047
CABG	18 (7.3)	0 (0.0)	18 (8.8)	0.049
HTN	92 (37.2)	19 (44.2)	73 (35.8)	0.300
DM	81 (32.8)	10 (23.3)	71 (34.8)	0.143
CVA	24 (9.7)	1 (2.3)	23 (11.3)	0.089
CKD	43 (17.4)	1 (2.3)	42 (20.6)	0.004
LC	16 (6.5)	3 (7.0)	13 (6.4)	1.000
TPL	17 (6.9)	2 (4.7)	15 (7.4)	0.744
**Laboratory data on day of arrest**				
Initial pH	9.2 (3.9)	7.19 (0.18)	7.14 (0.20)	0.172
Initial lactate (mmol/L*)	8.8 (6.2–12.1)	8.0 (5.4–11.6)	8.9 (6.4–12.1)	0.299
Hb (g/dL*)	9.7 (7.5–12.3)	9.2 (7.6–11.6)	9.9 (7.4–12.5)	0.624
PLT (× 10^3^/μL*)	135 (74–195)	154 (81–214)	131 (68–192)	0.178
Albumin (g/dL)	2.36 (0.79)	2.28 (0.79)	2.38 (0.79)	0.455
Creatinine (mg/dL*)	1.32 (1.00–2.00)	1.15 (0.86–1.30)	1.42 (1.05–2.15)	0.002
K (mmol/L*)	4.3 (3.7–5.0)	4.3 (3.6–5.5)	4.3 (3.8–5.0)	0.818
CRP (mg/dL*)	2.43 (0.21–6.00)	1.65 (0.15–6.96)	2.52 (0.22–5.93)	0.698

*BMI* Body mass index, *CPC* Cerebral performance category, *AMI* Acute myocardial infarction, *HF* Heart failure, *PCI* Percutaneous coronary intervention, *CABG* Coronary artery bypass graft, *HTN* Hypertension, *DM* Diabetes mellitus, *CVA* Cerebrovascular accident, *CKD* Chronic kidney disease, *LC* Liver cirrhosis, *TPL* Transplantation.

*Median (interquartile range), otherwise mean (SD).

**Figure 1:**
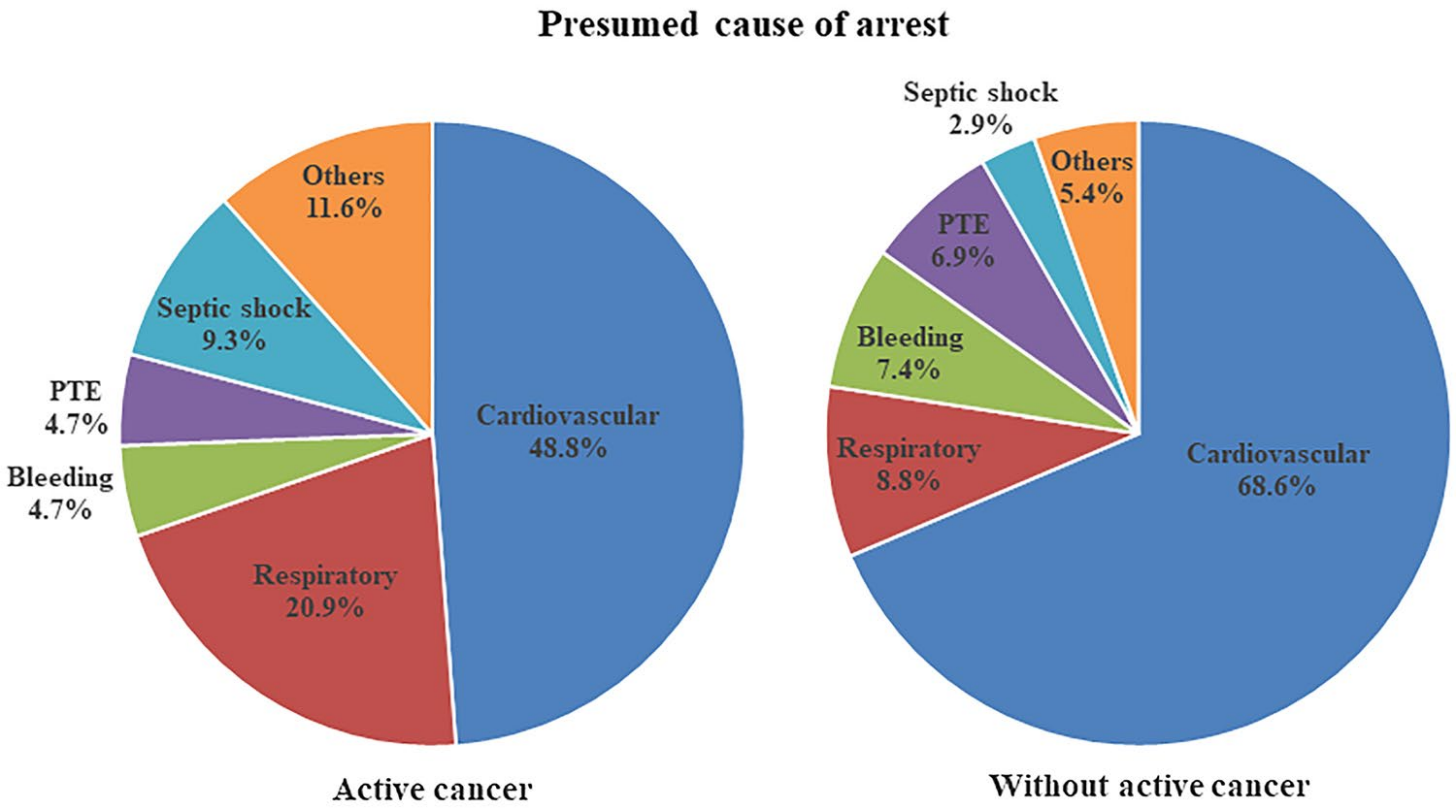
Presumed causes of cardiac arrest in patients with and without active cancer *PTE* Pulmonary thromboembolism.

The ECPR-related variables, including the location of arrest, presumed cause of arrest, total resuscitation time, and post-ECPR management, are shown in Table 2. Among the patients with active cancer, the ward was the most common location of arrest, but the ICU was most common in cancer-free patients. The proportion of cardiovascular aetiologies was significantly higher in cancer-free patients, while patients with active cancer had a higher proportion of respiratory aetiologies (Fig. 1). The total resuscitation time, total epinephrine dose, and the proportion of initial shockable rhythm, TTM, PCI, CABG, valve surgery, embolectomy, and use of vasopressors were not significantly different between the two groups. However, the cancer-free patients had a longer ECMO duration and received more renal replacement therapy.

**Table 2:** ECPR-related variables.

Variables	Total (n = 247)	Patients with active cancer (n = 43)	Patients without active cancer (n = 204)	*P*–value
**Location of arrest (%)**				
ICU	86 (35.0)	11 (25.6)	75 (36.9)	0.162
Ward	66 (26.8)	16 (37.2)	50 (24.6)	0.087
Operation room	13 (5.3)	6 (14.0)	7 (3.4)	0.013
Emergency room	49 (19.9)	5 (11.6)	44 (21.7)	0.137
Laboratory	21 (8.5)	3 (7.0)	18 (8.9)	1.000
Other	11 (4.5)	2 (4.7)	9 (4.4)	1.000
**Presumed cause of arrest (%)**				
Cardiovascular etiology	161 (65.2)	21 (48.8)	140 (68.6)	0.013
Ischemic heart disease	75 (30.4)	8 (18.6)	67 (32.8)	
Primary arrhythmia	42 (17.0)	7 (16.3)	35 (17.2)	
Heart failure	35 (14.2)	5 (11.6)	30 (14.7)	
Myocarditis/endocarditis	6 (2.4)	0 (0.0)	6 (2.9)	
Acute aortic syndrome	3 (1.2)	1 (2.3)	2 (1.0)	
Respiratory	27 (10.9)	9 (20.9)	18 (8.8)	0.030
Bleeding	17 (6.9)	2 (4.7)	15 (7.4)	0.744
Pulmonary embolism	16 (6.5)	2 (4.7)	14 (6.9)	0.745
Septic shock	10 (4.0)	4 (9.3)	6 (2.9)	0.076
Others	16 (6.5)	5 (11.6)	11 (5.4)	0.166
**CPR-related**				
Total resuscitation time (min*)	21 (11–35)	24 (10–41)	21 (11–33)	0.619
Total epinephrine dose (mg*)	6 (3–10)	6 (2–11)	6 (3–10)	0.835
ECMO duration (hour*)	71.8 (11.1–151.0)	27.4 (4.2–92.7)	75.3 (13.5–169.3)	0.005
Initial shockable rhythm (%)	50 (20.2)	7 (16.3)	43 (21.1)	0.477
**Post-ECPR management (%)**				
TTM	20 (8.1)	4 (9.3)	16 (7.8)	0.759
PCI	49 (19.8)	5 (11.6)	44 (21.6)	0.137
CABG	27 (10.9)	2 (4.7)	25 (12.3)	0.185
TPL	24 (9.7)	0 (0.0)	24 (11.8)	0.011
Valve surgery	7 (2.8)	1 (2.3)	6 (2.9)	1.000
Embolectomy	7 (2.8)	1 (2.3)	6 (2.9)	1.000
Renal replacement therapy	148 (59.9)	20 (46.5)	128 (62.7)	0.048
Vasopressor	225 (91.1)	40 (93.0)	185 (90.7)	0.775

*ICU* Intensive care unit, *ECMO* Extracorporeal membrane oxygenation, *TTM* Targeted temperature management, *PCI* Percutaneous coronary intervention, *CABG* Coronary artery bypass graft, *TPL* Transplantation.

*Median (interquartile range), otherwise mean (SD).

### Good neurologic outcome at 6 months (primary outcome)

**Figure 2:**
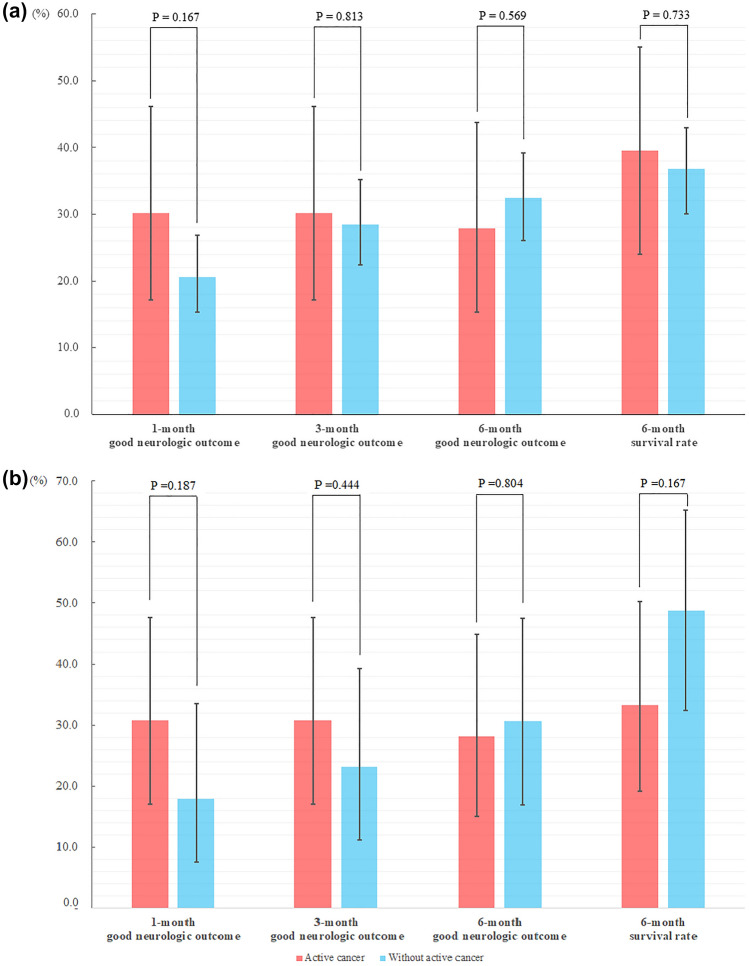
(**a**) Rates of good neurologic outcome at 1-, 3-, and 6-months, and the 6-month survival rates in patients with and without active cancer, (**b**) Analysis following matching with age, sex, comorbidities, presumed cause of arrest, and total resuscitation time.

With regard to good neurologic outcome at 6 months from the day of arrest, 12 patients with active cancer (27.9%; 95% CI, 15.3%–43.7%) had a good neurologic status, , whereas 66 patients without cancer (32.4%; 95% CI, 26.0%–39.2%) were found to have a good neurologic status (*P* = 0.569) (Fig. 2a). Results after matching were in the same manner with the results without matching, in which patients with active cancer (28.2%; 95% CI, 15.0%–44.9%) showed a good neurologic status and patients without cancer (30.8%; 95% CI, 17.0%–47.6%) had a good neurologic status (*P* = 0.804) (Fig. 2b).

### Good neurologic outcomes at 1 and 3 months, and the 6-month survival rate (secondary outcomes)

Likewise, regardless of the matching there was no significant difference between patients with active cancer and those without with regard to the rate of good neurologic outcomes at 1 month (without matching; 30.2% vs. 20.6%, *P* = 0.167, with matching; 30.8% vs. 17.9%, *P* = 0.187) and 3 months (without matching; 30.2% vs. 28.4%, *P* = 0.813, with matching; 30.8% vs. 23.1%, *P* = 0.444) (Fig. 2). However, by comparing the primary outcome, the rate of good neurologic outcomes in patients with active cancer was shown to decrease over time, whereas the rate in cancer-free patients steadily increased.

**Figure 3:**
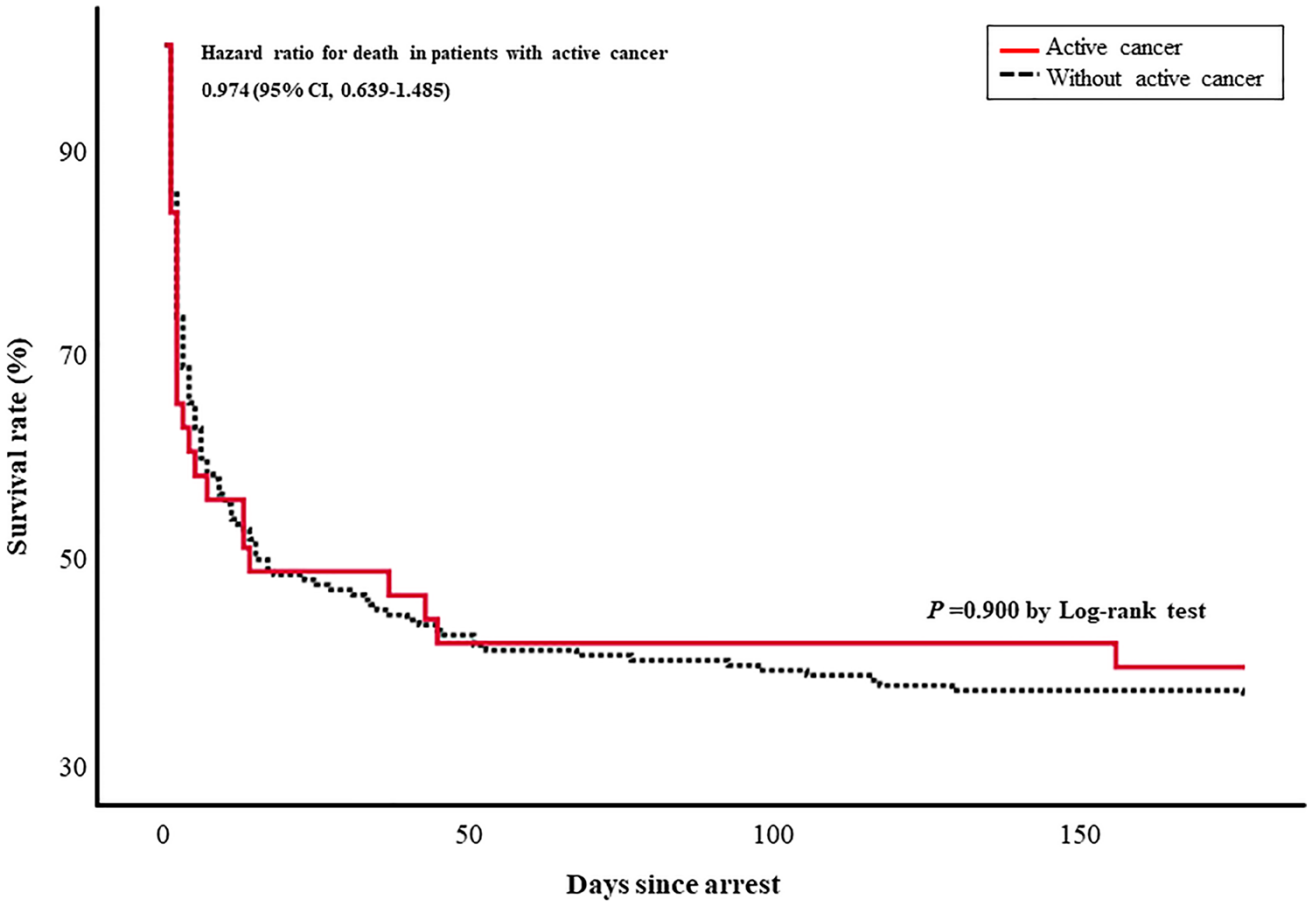
Kaplan–Meier survival curves over 6 months in patients with and without active cancer.

Seventeen patients with active cancer and 75 cancer-free patients survived until 6 months after the day of the arrest (39.5%; 95% CI, 24.0%–55.0% and 36.8%; 95% CI, 30.0%–43.0%, respectively); there was no significant difference between the two groups (*P* = 0.900 by log-rank test and hazard ratio 0.974; 95% CI, 0.639–1.485) (Figs. 2 and 3).

### Good neurologic outcome at 6 months based on the cause of arrest

Acute coronary syndrome as a presumed cause of arrest showed the highest good neurologic outcomes at 6 months in both groups. Over 30% of cancer-free patients with presumed cardiac causes had good neurologic outcomes at 6 months; however, patients with active cancer having presumed cardiac aetiologies had variable outcomes, ranging from 14 to 50%. Other causes of arrest, such as acute aortic syndrome, bleeding, and pulmonary thromboembolism, had lower rates of good neurologic outcome compared to cardiac causes (Supplementary Fig. 2).

### Association between active cancer and good neurologic outcome at 6 months

After dichotomizing patients into two groups, with and without good neurologic outcome at 6 months, univariable logistic regression analyses demonstrated that the presence of active cancer was not associated with good neurologic outcome at 6 months. Furthermore, multivariable logistic regression analysis, using the presence of active cancer and variables with *P*-values < 0.1, showed that the prearrest CPC score, initial shockable rhythm, total resuscitation time, and initial lactate were independently associated with 6-month good neurologic outcome, whereas the presence of cancer was also shown to have no association with 6-month good neurologic outcome (Table 3).

**Table 3:** Univariable and multivariable logistic regression analyses regarding 6-month good neurologic outcome.

Variable	Univariable logistic regression				Multivariable logistic regression			
	OR	95% CI		*P*-value	OR	95% CI		*P*-value
		Lower	Upper			Lower	Upper	
Age (years)	0.991	0.973	1.009	0.335				
Male sex	2.147	1.107	4.164	0.024*	1.912	0.905	4.042	0.090
Prearrest CPC score	9.753	1.282	74.231	0.028*	8.823	1.119	69.536	0.039*
Active cancer	0.809	0.391	1.676	0.569	0.803	0.342	1.882	0.613
Initial shockable rhythm	2.444	1.293	4.622	0.006*	2.910	1.367	6.194	0.006*
Total resuscitation time (min)	0.966	0.948	0.984	< 0.001*	0.964	0.943	0.986	0.001*
Initial pH	13.758	2.905	65.161	0.001*	2.675	0.320	22.342	0.364
Initial lactate (mmol/L)	0.852	0.787	0.922	< 0.001*	0.893	0.812	0.983	0.021*
Creatinine (mg/dL)	0.940	0.782	1.130	0.509				

*Statistically significant.

*OR* Odds ratio, *CI* Confidence interval, *CPC* Cerebral performance category.

## Methods

### Data sources

We retrospectively reviewed the electronic medical records of all consecutive adult patients in the ECMO registry from January 2015 to December 2019 in Asan Medical Center, which serves as a tertiary referral centre. The ECMO registry is a database of adult patients who received ECMO regardless of the type. The current study protocol was approved by the Institutional Review Board of Asan Medical Center, and the requirement for informed consent was waived because of the retrospective nature of the analyses. All methods were carried out in accordance with relevant guidelines and regulations.

### Study population

Among all consecutive adult patients from the ECMO registry from January 2015 to December 2019, we first excluded patients treated with ECMO in a non-arrest situation, those who received veno-veno ECMO, and those who received ECPR for out-of-hospital cardiac arrest (OHCA). The remaining patients with IHCA who were treated with ECPR were reviewed and divided into two groups based on the presence or absence of active cancer. Active cancer was defined according to the Haemostasis and Malignancy Scientific and Standardization Committee definition as follows: (1) Cancer diagnosed within the previous 6 months; (2) recurrent, regionally advanced, or metastatic cancer; (3) cancer for which treatment had been administered within the previous 6 months; and (4) haematological cancer that is not in complete remission^22^.

### Outcomes

The primary outcome was a good neurologic status at 6 months from the day of IHCA, defined as 1 or 2 on the Cerebral Performance Category (CPC) score, at which stage patients are able to perform daily activities independently and are able to work in a sheltered environment. The secondary outcomes were a good neurologic status, defined in the same manner as in the primary outcome, at 1 and 3 months from the day of arrest. The survival rate at 6-month was also evaluated. In addition, by investigating the presumed cause of arrest, we compared the rate of 6-month good neurologic status between the two groups.

### Statistical analysis

Data were first tested for normality. Continuous variables with normal distributions are presented as mean ± standard deviation, and those with non-normal distributions are expressed as median ± interquartile range (IQR). Categorical variables are presented as n (%). Continuous variables were compared using the Student’s t-test and Mann–Whitney U test as appropriate, and categorical variables were compared using the chi-square test and Fisher’s exact test accordingly.

With regard to good neurologic outcomes, the CPC score was dichotomized into two types, good (CPC 1 or 2) and poor (CPC 3–5) neurologic status, expressed as percentage with 95% confidence interval, and compared using a chi-square test. Propensity matching was also conducted with age, gender, comorbidities, presumed cause of arrest, and total resuscitation time, followed by comparing between those with cancer and those without. The cumulative survival rates of the two groups are presented with Kaplan–Meier curves and were compared by the log-rank test and the hazard ratio.

Univariable and multivariable logistic regression analyses about good neurologic outcome at 6 months were also performed to determine whether active cancer is independently associated with 6-month good neurologic outcome. All statistical analyses were performed using IBM SPSS Statistics V21.0 (SPSS Inc, Chicago, IL). *P*-values < 0.05 were considered statistically significant.

### Ethics approval and consent to participate

This study was approved by the Research Ethics Committee of Asan Medical Center (2021–0115) which waived the requirement for patient informed consent.

## Discussion

In this study, we aimed to establish the short- and long-term good neurologic outcomes for patients who undergo IHCA and receive ECPR, specifically those for patients with active cancer, by comparing them with cancer-free patients. We aimed to show that patients with active cancer should not be excluded from receiving ECPR solely because of the presence of active cancer.

Of the 247 patients included in this study, 43 patients had active cancer, and the remaining 204 patients had no cancer. Respiratory causes of IHCA were higher in patients with active cancer, and cardiovascular causes were higher in cancer-free patients. Comparing patients with active cancer and patients without cancer, we found no significant difference in good neurologic outcomes at 1 month (30.2% vs. 20.6%), 3 months (30.2% vs. 28.4%), and 6 months (27.9% vs. 32.4%) from the day of arrest. Likewise, the survival rate at 6 months from the day of arrest was not significantly different between the two groups (*P* = 0.900).

No previous study has examined the good neurologic outcomes and survival rate of patients with active cancer who suffer IHCA and receive ECPR. Unlike traditional consensus, our study is considered to provide evidence for the implementation of ECPR to broaden its inclusion criteria, especially patients with active cancer who have been growing in number over the past few decades.

Previous studies have examined the survival to hospital discharge between patients with and without cancer^14,23^. Both of the two previous studies showed that patients with cancer had a lower survival rate than those without (31% vs. 46%), which is inconsistent with the results of our study, although Kang et al.^23^ investigated patients who underwent OHCA. Lower use of post-cardiac arrest management, such as angiography, PCI, TTM, and a smaller proportion of initial shockable rhythm in patients with cancer, was considered to be the major reason for the differences in survival rates; in support of this explanation, previous studies demonstrated that an initial shockable rhythm was associated with a higher rate of PCI due to its high likelihood of cardiac origin, which PCI could benefit^24,25^. However, our study showed no significant difference in the proportion of initial shockable rhythm and post-cardiac arrest management such as TTM, PCI, CABG, valve surgery, embolectomy, and use of vasopressors, except for renal replacement therapy and heart transplantation. Considering our results, it is reasonable to consider that offering proper post-cardiac arrest management, including ECPR, would increase the proportion of good neurologic outcomes and the survival rate of patients with active cancer to a level similar to that of patients without cancer. This is supported by the study of Champigneulle et al.^26^, who demonstrated that the 6-month survival rate was significantly different in the unmatched comparison of patients with and without malignancies who underwent cardiac arrest, but not in the matched comparison, although they did not focus on patients who received ECPR.

With regard to the rate of good neurologic outcomes over time, the rate in patients with active cancer decreased from 1 to 6 months following arrest, but cancer-free patients showed a steady increase in percentage. Although it seems natural that the rate of good neurologic outcome tend to decrease over follow-up, like the rate of patients with active cancer, as shown in a study by Meng-Rui et al.^27^, the result of cancer-free patients in our study contradicts previous studies^5,28^. This can be explained by the rate of heart transplantation that was performed in cancer-free patients. Generally, ECMO is considered as a bridge to heart transplantation for patients with decompensated heart failure^29^, which has a good long-term favourable outcome. As cancer-free patients had undergone more heart transplantation surgery than patients with active cancer (11.8% vs. 0.0%), this might have affected the long-term favourable neurologic outcomes. In addition, cardiovascular aetiologies that are found more frequently in cancer-free patients compared to patients with active cancer are also considered good prognostic factors.

The limitations of the current study mainly relate to its retrospective nature; with retrospective studies, there is always the possibility that selection bias may have influenced the results, particularly in the indication of ECPR in patients with active cancer. Secondly, the relatively small sample size led to the failure to demonstrate an association between the presence of active cancer and outcomes. A further non-inferiority trial will be needed to confirm our results. Thirdly, it was done by a single centre, which does not guarantee the adaptation of the generalized population. As a different environment could also make the result different, the results, therefore, should be interpreted with caution. Lastly, in terms of cost–benefit of ECPR in patients with active cancer, quality-adjusted life year (QALY) should be considered since it is not effective when malignancy itself which truncates patients’ life expectancy outweighs the benefit of ECPR which might increase QALY to some extent. However, we did not conduct cost–benefit analysis due to lack of data about the expenses for ECPR. Therefore, further investigation is needed in light of the total cost for ECPR and the benefit of QALY.

## Conclusion

Patients with active cancer who suffer IHCA and undergo ECPR show similar 6-month good neurologic outcome and survival rate in comparison with patients without cancer. Therefore, ECPR should not be excluded as a treatment option solely on the basis of the presence of active cancer.

## Supplementary Information

Supplementary Information.
