# Supplementary material for: The feasibility of extracorporeal cardiopulmonary resuscitation for patients with active cancer who undergo in-hospital cardiac arrest
Source: Sci Rep. 2022 Jan 31;12:1653. doi: 10.1038/s41598-022-05786-8 (PMC8803995; doi:10.1038/s41598-022-05786-8)
Supplement: Supplementary file 1 — Supplementary Information. [file 41598_2022_5786_MOESM1_ESM.docx]

**Supplementary figure 1**. Details in patients with cancer who undergone extracorporeal cardiopulmonary resuscitation (ECPR)


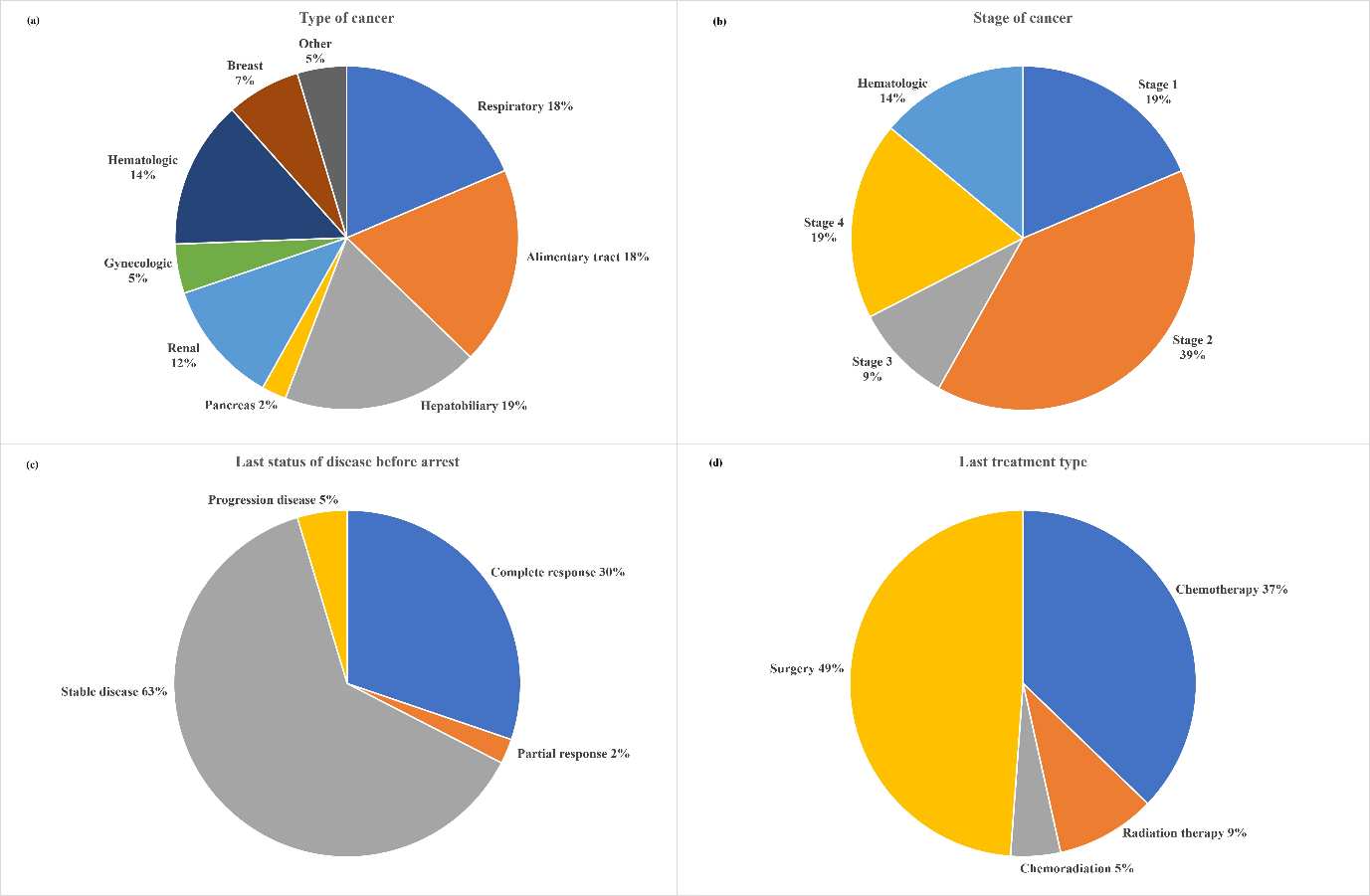


**Supplementary figure 2.** Rate of 6-month good neurologic outcome based on the cause of arrest


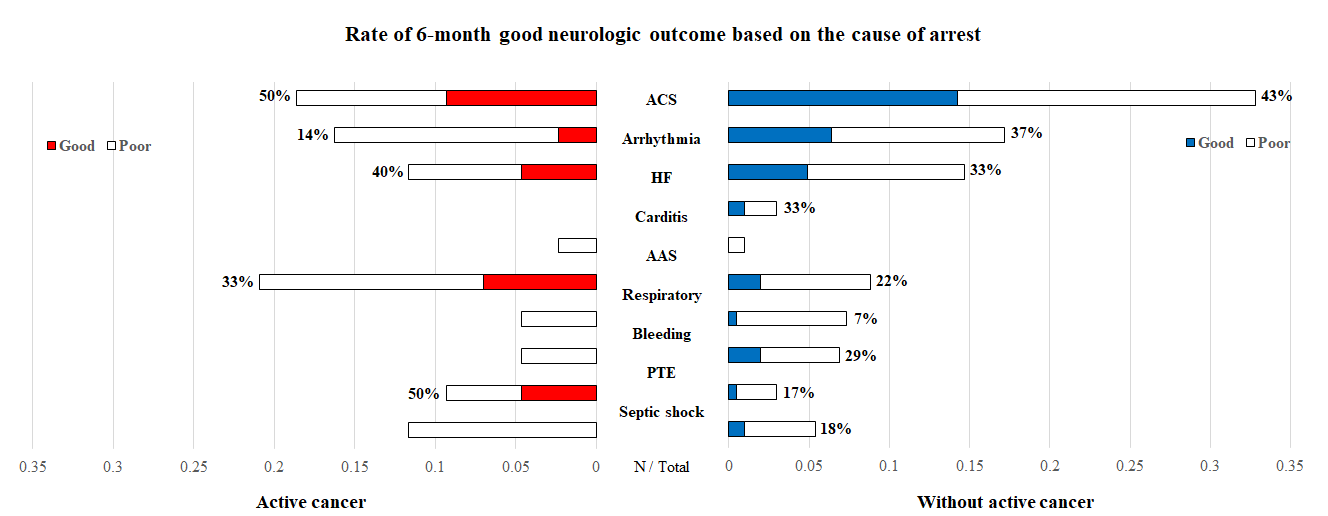


*ACS*; Acute coronary syndrome, *HF*; Heart failure, *AAS*; Acute aortic syndrome, *PTE*; Pulmonary thromboembolism
